# Supplementary material for: Combining residual U-Net and data augmentation for dense temporal segmentation of spike wave discharges in single-channel EEG
Source: Sci Rep. 2026 Jul 21;16:33171. doi: 10.1038/s41598-026-62064-7 (PMC13683039; doi:10.1038/s41598-026-62064-7)
Supplement: Supplementary file 1 [file 41598_2026_62064_MOESM1_ESM.pdf]

Table 6. AugUNet1D Architecture. BN = Batch Normalization; MP = MaxPool1d; CT = ConvTranspose1d. Residual projection uses a 1×1 convolution to match channel dimensions. Kernel sizes are 5 for encoder conv blocks and 3 for decoder conv blocks unless noted.

Stage	Block	Layer	In Channels	Out Channels	Kernel	Operations
**Encoder**	Enc Block 1	Conv1	1	64	5	Conv1d, ReLU
		Conv2	64	64	5	Conv1d, BN, ReLU
		Residual + Pool	1	64	1	Conv1d (1×1), MaxPool1d(2)
	Enc Block 2	Conv1	64	128	5	Conv1d, BN, ReLU
		Conv2	128	128	5	Conv1d, BN, ReLU
		Residual + Pool	64	128	1	Conv1d (1×1), MaxPool1d(2)
	Enc Block 3	Conv1	128	256	5	Conv1d, BN, ReLU
		Conv2	256	256	5	Conv1d, BN, ReLU
		Residual + Pool	128	256	1	Conv1d (1×1), MaxPool1d(2)
	Enc Block 4	Conv1	256	512	5	Conv1d, BN, ReLU
		Conv2	512	512	5	Conv1d, BN, ReLU
		Residual + Pool	256	512	1	Conv1d (1×1), MaxPool1d(2)
**Bottleneck**	Bottom Block	Conv1	512	1024	3	Conv1d, BN, ReLU
		Conv2	1024	1024	3	Conv1d, BN, ReLU
		Residual	512	1024	1	Conv1d (1×1)
**Decoder**	Dec Block 1	Upsample	1024	1024	2	ConvTranspose1d(stride=2)
		Conv1	1536	512	3	Conv1d, BN, ReLU
		Conv2 + Residual	512	512	3	Conv1d, BN, ReLU; Conv1d (1×1)
	Dec Block 2	Upsample	512	512	2	ConvTranspose1d(stride=2)
		Conv1	768	256	3	Conv1d, BN, ReLU
		Conv2 + Residual	256	256	3	Conv1d, BN, ReLU; Conv1d (1×1)
	Dec Block 3	Upsample	256	256	2	ConvTranspose1d(stride=2)
		Conv1	384	128	3	Conv1d, BN, ReLU
		Conv2 + Residual	128	128	3	Conv1d, BN, ReLU; Conv1d (1×1)
	Dec Block 4	Upsample	128	128	2	ConvTranspose1d(stride=2)
		Conv1	192	64	3	Conv1d, BN, ReLU
		Conv2 + Residual	64	64	3	Conv1d, BN, ReLU; Conv1d (1×1)
**Classifier**	Output	Conv	64	1	1	Conv1d (1×1), Sigmoid
